# Australia’s ‘silent pandemic’ of diabetes complications: where do feet stand in this pandemic?

**DOI:** 10.1186/1757-1146-6-S1-O25

**Published:** 2013-05-31

**Authors:** Peter A Lazzarini, Joel M Gurr, Joseph R Rogers, Andrew Schox, Shan M Bergin

**Affiliations:** 1 Allied Health Research Collaborative, Metro North Hospital & Health Service, Queensland Health, Brisbane, Queensland, 4032, Australia; 2 School of Clinical Sciences, Queensland University of Technology, Brisbane, Queensland, 4059, Australia; 3 Podiatry Department, Royal Perth Hospital, South Metropolitan Health Service, WA Health, Perth, Western Australia, 6000, Australia; 4 John Morris Diabetes Centre, Launceston General Hospital, Launceston, Tasmania, 7250, Australia; 5 The Perth Foot & Ankle Clinic, Perth, Western Australia, 6154, Australia; 6 Diabetic Foot Unit and High Risk Foot Service, Dandenong Hospital, Southern Health, Melbourne, Victoria, 3175, Australia

## Background

Diabetes is Australia’s leading cause of kidney failure, blindness (under 60yo), and amputation, plus, causes significant cardiovascular disease. Australia’s diabetes amputation rate has increased by 30% in the last decade and is one of the worst in the developed world, yet other Australian diabetes complication outcomes have improved. This paper aims to compare the national burden of disease for the four major diabetes-related complications and the availability of government funding to combat these complications, in order to determine where diabetes foot disease ranks in Australia.

## Methods

Electronic databases, government and health websites were searched for papers (1995 – 2012) reporting Australian national diabetes-related complication numbers, incidence or prevalence rates, burden of disease, economic costs and program funding. Publications reviewed included epidemiological, health economic, evidence-based guidelines, government, Medicare and Pharmaceutical Benefits Scheme reports.

## Results

Foot disease ranked second in numbers affected, deaths, cost per episode and overall burden of disease of the four diabetes complications in Australia. However, 50% of the national evidence-based diabetic foot disease guideline recommendations are funded via Medicare, compared to 100% of other national diabetes complication guideline recommendations. Furthermore, foot disease ranked last for additional program funding.

## Conclusions

Findings suggest foot disease is the second leading cause of burden of disease, yet receives the least available government funding of the four major diabetes complications in Australia. This low level of clinical funding may be a major factor in Australia’s poor end stage foot outcomes (amputation rates) compared to other diabetes end stage outcomes.

